# Effectiveness of nicotine salt vapes, cytisine, and a combination of these products, for smoking cessation in New Zealand: protocol for a three-arm, pragmatic, community-based randomised controlled trial

**DOI:** 10.1186/s12889-023-16665-w

**Published:** 2023-09-11

**Authors:** Natalie Walker, Amanda Calder, Joanne Barnes, George Laking, Varsha Parag, Chris Bullen

**Affiliations:** 1 School of Population Health, National Institute for Health Innovation, The University of Auckland, Private Bag 92019, Auckland, 1142 New Zealand; 2 School of Pharmacy, The University of Auckland, Private Bag 92019, Auckland, 1142 New Zealand; 3 Department of Molecular Medicine and Pathology, The University of Auckland, Private Bag 92019, Auckland, 1142 New Zealand

**Keywords:** Cytisine, e-cigarettes, Nicotine salt, Smoking cessation, Randomised, Trial

## Abstract

**Background:**

Combining short-acting nicotine replacement therapy with varenicline increases smoking cessation rates compared with varenicline alone, but not all people tolerate these medications or find them helpful. We aim to investigate the therapeutic potential of an analogous combination, by evaluating the effectiveness, safety, and acceptability of combining nicotine salt e-cigarettes with cytisine, compared to nicotine salt e-cigarettes or cytisine only, on smoking abstinence at six months.

**Methods:**

A pragmatic, community-based, investigator-blinded, randomised superiority trial design will be utilised. Eligible participants will be people who smoke daily (*N* = 800, 90% power) from throughout New Zealand, who are: aged ≥ 18 years, motivated to quit in the next two weeks, able to provide online consent, willing to use e-cigarettes and/or cytisine, and have daily access to a mobile phone. Recruitment will utilise multi-media advertising. Participants will be randomised (3:3:2 ratio) to 12 weeks of: 1) e-cigarettes (closed pod system, 3% nicotine salt, tobacco flavour) plus cytisine; 2) e-cigarettes alone, or 3) cytisine alone. All groups will receive a six-month, text-message-based behavioural support programme. The primary outcome is self-reported, biochemically verified, continuous abstinence at six months post-quit date. Secondary outcomes, measured at quit date, then one, three, six, and 12 months post-quit date, include self-reported continuous abstinence, 7-day point prevalence abstinence, cigarettes smoked per day, withdrawal and urge to smoke, time to (re)lapse, treatment use and compliance, treatment crossover, dual-use, use of other cessation products, change in e-cigarette products, continuation of product use, acceptability, change in health state, health-related quality of life, change in body mass index, adverse events, and cost per quitter.

**Discussion:**

Pragmatic trials are of particular value as they reflect the ‘real world’ impact of interventions. The trial will provide some of the first evidence on the effectiveness of combining nicotine salt e-cigarettes with cytisine for smoking cessation, in a country with strong tobacco control policy. Findings will be incorporated into relevant systematic reviews, informing practice and policy.

**Trial registration:**

NCT05311085 ClinicalTrials.gov. Registered 5th April, 2022.

## Introduction

The prevalence of smoking in New Zealand (NZ) continues to decline across all population subgroups, however, there remain marked disparities in the prevalence of daily smoking for specific subgroups. For example, in 2021/22 8% of people in NZ aged ≥ 15 years smoked daily, although smoking prevalence was higher in indigenous Māori and Pacific people (20% and 18% respectively, compared with 7% European/Other and 3% Asian) and people living in the most deprived areas of NZ (17%, compared with 5% in the least deprived areas) [1].

The current standard for smoking cessation treatment in NZ combines behavioural support strategies with pharmacotherapy (combination nicotine replacement therapy [NRT], varenicline, bupropion, or nortriptyline) [2, 3]. However, only about one-third of people who smoke quit smoking with the use of NRT or varenicline [4–6], and uptake of these products in NZ is low (despite products being subsidised by the NZ government) [7–9]. Also, a third of people who smoke are positive responders to NRT, as low-dose NRT does not fully saturate all nicotinic acetylcholine receptors (nAChRs). Furthermore, although varenicline primarily targets the alpha4-beta2 and alpha7 nAChRs, as a partial agonist it does not fully saturate the receptors, so does not completely replace the dopaminergic effect of smoking. Although not currently recommended by the NZ smoking cessation guidelines [2, 3], combining varenicline and NRT can increase smoking abstinence rates compared with use of varenicline alone (Continuous abstinence [CA] ≥ six months**:** Relative Risk [RR] = 1.62, 95% Confidence Interval [CI] 1.18–2.23, two trials, *N *= 787), with no significant increase in the frequency of adverse events (such as nausea, insomnia, and vivid dreams) [10]. Two other effective smoking cessation interventions exist that are more acceptable to users than NRT and varenicline, namely cytisine and e-cigarettes (nicotine vapes) [8, 9].

Cytisine is a plant-derived alkaloid with a long history of use in Central and Eastern Europe as a smoking cessation medication [11]. Cytisine is structurally similar to nicotine and, like varenicline, acts as a partial agonist at nAChRs [11]. A pragmatic community-based non-inferiority trial (*N* = 679) found 12 weeks’ treatment with oral cytisine to be at least as effective as 12 weeks treatment with varenicline in supporting verified smoking abstinence at six months (12.1% vs 7.9% respectively, Risk Difference = 4.3, 95% CI -0.22–8.79) in NZ Māori or family of Māori, but with significantly fewer self-reported adverse events (AEs) observed in the cytisine group (Incidence Rate Ratio [IRR] = 0.56, 95% CI 0.49–0.65, *p*< 0.001) [9]. A second non-inferiority trial failed to demonstrate non-inferiority for 25 days of cytisine compared to 12 weeks of varenicline for six-month smoking abstinence (verified CA: 11.7% vs 13.3% respectively) in 1,452 Australians who smoked [12]. Like the NZ trial, the Australian study demonstrated significantly fewer self-reported AEs in the cytisine group (IRR = 0.88, 95% CI 0.81–0.95, *p* = 0.002) [12]. Importantly, in both trials nausea occurred less frequently in the cytisine group than in the varenicline group, likely due to cystine’s lower potency at 5-hydroxytryptamine receptors (5-HT_3_) [13]. An additional advantage of cytisine over varenicline is the price difference between the two drugs in markets where they are currently approved [14], and the much lower cost per quality-adjusted life-years for cytisine [15]. Furthermore, modelling indicates cytisine may be more cost-effective than varenicline [16, 17]. Currently, cytisine is not an approved medicine in New Zealand.

E-cigarettes replace some of the nicotine people obtain from tobacco. E-cigarettes (with or without nicotine) also help address some of the behavioural hand-to-mouth actions associated with smoking [18, 19]. A Cochrane systematic review on e-cigarettes for smoking cessation found nicotine e-cigarettes were superior to NRT (Six studies, *N* = 2,378, RR = 1.63, 95% CI 1.30–2.04) and nicotine-free e-cigarettes (Five studies, *N*= 1,447, RR = 1.94, 95% CI 1.21–3.13) at helping people to quit smoking for at least six months [20]. The review also concluded that the overall incidence of serious AEs was low, with no clear evidence of harm from nicotine e-cigarettes based on available data [20]. The Cochrane review highlighted the need for more trials on the effectiveness of e-cigarettes, particularly of the new nicotine salt pod e-cigarettes given their faster nicotine delivery [20]. Evidence from earlier versions of this review, and the findings of two NZ e-cigarette trials undertaken by our team and included in the review (total *N* = 1,781 [7, 21]), have informed the NZ government’s decision to endorse e-cigarettes as an acceptable product to support switching away from tobacco smoking (https://vapingfacts.health.nz/), within the bounds of restrictions on advertising, sponsorship, packaging, flavourings, and the provision of health information [22]. E-cigarettes (with or without nicotine) are widely available in NZ, through supermarkets, specialist 'vape' shops, on-line retailers and pharmacies. National survey data for 2021/22 found 8% of people in NZ aged ≥ 15 years vaped daily, with vaping rates higher in indigenous Māori and Pacific people (18% and 17% respectively, compared with 8% European/Other and 6% Asian) and people living in the most deprived areas of NZ (10%, compared with 6% in the least deprived areas) [1].

The effectiveness of the combined use of cytisine and nicotine e-cigarettes on smoking abstinence has not yet been investigated but may provide a way to further maximise the chances of quitting smoking. In NZ it is standard practice to provide behavioural support as an adjunct to any pharmacological cessation support for people wanting to quit smoking [2, 3], so it would be appropriate to add such support to nicotine e-cigarettes and cytisine to further boost quitting. A Cochrane systematic review reported that automated text messaging behavioural support is more effective than minimal smoking cessation support (Abstinence ≥ six months**:** RR = 1.54, 95% CI 1.19–2.00, 13 trials, *N* = 14,133), and that combining automated text-based behavioural support (TBS) with a smoking cessation intervention is more effective than the use of a smoking cessation intervention alone (Abstinence ≥ six months**:** RR = 1.59, 95% CI 1.09–2.33, four trials, *N *= 997) [23]. Building on this existing research, and considering the value of ‘real-world’ evidence, we designed a single-blind, pragmatic, three-arm, parallel-group, community-based randomised trial to evaluate the effectiveness, safety and acceptability of combining cytisine with nicotine e-cigarettes and TBS, compared to cytisine with TBS or nicotine e-cigarettes with TBS only, on six-month smoking abstinence. We hypothesize that combination treatment is more effective than monotherapy at increasing quit rates at six months post-quit date.

## Methods

### Study population

People who live in NZ and smoke tobacco every day.

### Eligibility criteria

Eligible participants are those aged ≥ 18 years, motivated to quit smoking within the next two weeks, able to provide online consent, have daily access to a mobile telephone that can text, and have access to the internet via a computer or smartphone. Participants must also be willing to use either of the intervention products to help quit smoking.

Pregnant or breastfeeding women or women who are trying to become pregnant in the next three months will be excluded, as will be people currently using smoking cessation medication (including daily use of an e-cigarette in the last month), enrolled in another cessation programme or trial, with known hypersensitivity to cytisine or nicotine e-cigarettes or with history of severe allergy and/or poorly controlled asthma. Participants will be excluded if they have a strong preference to use or not use either product in their quit attempt or who have another member of their household already enrolled in the study. Participants will also be excluded if they self-report moderate/severe renal impairment, are undergoing treatment for active/latent tuberculosis, have experienced a myocardial infarction, stroke, or severe angina within the previous two weeks, have uncontrolled high blood pressure (> 150 mmHg systolic, > 100 mmHg diastolic) or have a history of seizures. These exclusion criteria are based on the product insert for cytisine and are precautionary (evidence to support these exclusions is absent or limited) [11].

### Recruitment

Recruitment will be undertaken nationally using multi-media advertising, with targeted promotion to reach Māori, Pasifika and low socio-economic groups given their disproportionately higher smoking prevalence [1]. Participation in the trial will also be promoted via the national Quitline and community-based smoking cessation providers and general practitioners (GP).

Advertisements will direct potential participants to a study website where they can read the participant information sheet. A two-step-consent process will be used. First, interested participants will be asked for online consent to complete an online screening questionnaire to determine their eligibility for the trial and verify their contact details. Second, eligible and interested participants must provide online consent to enter the trial.

Baseline data will be collected via the online platform. Participants will then be asked to click the ‘randomisation’ button, whereupon they will be immediately randomised, informed of their allocated intervention, and automatically emailed a copy of the consent form and participant information sheet for their records. Their usual GP will also be automatically notified that their patient is enrolled in the trial.

### Randomisation, allocation concealment and sequence generation

Participants will be assigned a unique registration number allocated by a central computer, following details submitted via the website. Eligible participants will be randomised via computer (3:3:2 ratio) to one of three trial groups, using stratified block randomisation (block sizes of eight), and stratified by ethnicity (Māori, non-Māori). The randomisation sequence will be centrally managed and concealed until the point of randomisation.

### Blinding

This is a single-blind trial—participants will be aware of the intervention to which they have been allocated, and data collection by the trial research assistants includes questions specific to participants’ use of their allocated treatment. Except for the trial statistician (VP), the trial steering committee members will be blinded to treatment allocation until analyses are complete. If one or more of the study’s registered medical practitioners, or the study pharmacist, requires more details about a serious AE that requires unblinding, then they may request that the event be assessed by members of the trial’s independent Data Safety Monitoring Committee (DSMC), who will be unblinded by the project manager for the event.

### Study interventions

Participants will be randomised to one of three treatments: 1) a nicotine e-cigarette, 2) cytisine, or 3) a nicotine e-cigarette plus cytisine, for 12 weeks. Trial products will be couriered to participants immediately after randomisation (signature required for receipt). The courier pack includes information on how to use the allocated product(s), a copy of the participant information sheet and consent form, a wallet card summarising how to use the allocated treatment, what participants should do if they experience any adverse health events, and contact details for key members of the study team. The courier company will automatically notify the study centre once the courier pack has been delivered, which will trigger the scheduling of the ‘quit date’ follow-up call and the start of the TBS programme.

Participants will be advised to begin their treatment the day after they receive their courier pack. They will be asked to reduce their smoking ad libitum over the first four days of treatment. The fifth day of treatment will be the participants designated ‘quit date’. Participants will be called on their quit date to verify they quit on that day and to collect outcome data. If a participant states that they have not quit on the scheduled ‘quit date’, they will be given one chance to reset their quit date (to within the next seven days), with this date becoming their new ‘quit date’ (which triggers the scheduling of all subsequent follow-up calls). For participants who cannot be contacted, their ‘quit date’ will be set in the system as six days after receiving their courier pack. Participants will be told that even if they lapse back to smoking, they should continue using the treatment they have been allocated. Throughout the trial, participants will be free to take whatever medications they wish, except for those noted under the exclusion criteria above.

### ***Treatment group (cytisine only):***

Participants allocated cytisine will be instructed to follow the manufacturer’s 25-day dosing regimen [11]:

- days 1–3: one tablet (1.5 mg) every two hours through the waking day (six tablets/day)
- days 4–12: one tablet every 2.5 h (five tablets/day)
- days 13–16: one tablet every three hours (four tablets/day)
- days 17–20: one tablet every 4–5 h (three tablets/day)
- days 21–25: one tablet every six hours (two tablets/day)

In addition, between day 26 and the end of week 12, participants will be advised to take a maintenance dose of one cytisine tablet every six hours (two tablets/day), to match the treatment duration used in a previous NZ cytisine vs varenicline non-inferiority trial [9].

#### Treatment group (nicotine e-cigarette only)

Participants allocated a nicotine e-cigarette will be instructed to follow the manufacturer’s instructions for use, with *ad* *libitum use* over 12 weeks. The e-cigarette device used in the trial is the ‘UpOX’ closed pod system containing 3% nicotine salt (30 mg/mL). A tobacco flavour e-liquid will be provided as this is the most common flavour chosen by people who smoke when transitioning away from tobacco onto vapes. Participants will be advised that they should try and use only the product provided, but if they find that the nicotine strength is not sufficiently addressing their cravings (or the flavour is unpleasant) they may try alternative nicotine strengths or flavours—but at their own cost. The nicotine e-liquid supplied will be independently assessed by LabTech Scientific and Technical Services, Auckland, NZ, to verify the nicotine content is as labelled.

#### Treatment group (cytisine plus nicotine e-cigarette)

Participants allocated cytisine plus the e-cigarette will be instructed to follow the manufacturer’s instructions for use, as above.

#### Behavioural support (all groups)

All participants will receive the STOMP programme [24], an evidence-based text message smoking cessation advice, support, distraction and motivation programme to support individuals to quit smoking and maintain cessation. The programme includes two-way functionality to support individuals during cravings, is personally tailored, and will be delivered over a six-month period (five messages a day for six weeks, then three per week until the end of the 26th week – i.e., six-month follow-up). Text messages can be received even if the phone has no credit. Participants can opt-out of the service at any time by free texting back ‘STOP QUIT’.

## Baseline assessments

The following baseline variables will be collected (Table 1):

- Demographic information: date of birth, gender, ethnicity, height (self-reported), weight (self-reported), and level of education.
- Smoking history: age when started, number of cigarettes smoked per day, number of years as a regular smoker, number of previous attempts to give up smoking in past 12 months (and the longest time they stayed quit and the method used), type of cigarettes smoked per day (e.g. roll-your-own or factory-made) and pack size and how long a pack lasts (for roll-your-own users), and whether they had cut down the number of cigarettes they smoked in the past 12 months.
- Motivation to quit in the next two weeks: measured using a five-point Likert Scale, where 1 = very low motivation and 5 = very high.
- Level of cigarette dependence: determined using the Heaviness of Smoking Index [25], which is a two-item measure based on the number of cigarettes smoked per day and the time to first cigarette of the day (from the Fagerström Test for Cigarette Dependence) [26].
- Other smoking-related information: self-rated chance of giving up smoking for good this time (measured using a five-point Likert Scale, where 1 = extremely low and 5 = extremely high), and whether others in the household also smoke tobacco.
- Other vaping-related information: whether they live with people who vape nicotine and/or have friends who vape nicotine.
- General Health: self-reported shortness of breath, cough, asthma, chronic pain, Chronic Obstructive Pulmonary Disease (COPD), and current or history of mental health (Depression, schizophrenia or related disorder, anxiety, and/or other mental health concerns).
- Concomitant medication: types of medication currently used.
- Alcohol use: measured using the Alcohol Use Disorders Identification Test (AUDIT-C) [27].
- Health related quality of life: (HRQoL) measured using the NZ EQ-5D Tariff 2 [28].
- Signs and symptoms of nicotine withdrawal and urge to smoke: measured using the Mood and Physical Symptoms Scale (MPSS) [29].
- Cannabis: whether they had used cannabis for recreational or non–medical use in the last 12 months. After answering this question information will be provided on how to access free 24/7 support if participants have concerns about their cannabis use.

**Table 1:** Details of baseline and follow-up outcomes

	**Online**			**Call 1**	**Call 2**	**Call 3**	**Call 4–5**
**Timing Description**	**Week 0 Screening, Baseline and Randomisation**			**Quit date (QD) Endpoint**	**One month after QD Endpoint**	**Three months after QD Endpoint**	**Six and 12 months after QD Endpoint**
**Case Record Form (CRF)**	**CRF-A**	**CRF-B**	**CRF-R**	**C-QD**	**C-1**	**C-3**	**C-6, C-12**
**General data**							
Eligibility criteria	X						
Consent	X	X					
Age, gender, ethnicity, education		X					
Height		X					
Weight		X				X	X
Current medication^d^		X		X	X	X	X
Health state		X				X	X
Alcohol and cannabis use		X					
Pregnancy	X			X	X	X	X
**Smoking information**							
Type of tobacco smoked		X					
Cigarettes smoked per day^b^		X		X	X	X	X
Age started		X					
Years smoked		X					
Time to first cigarette upon waking		X					
Household smoking		X					
Around others that use e-cigarettes		X					
Previous quit attempts and method		X					
Motivation to quit		X					
Nicotine withdrawal and urge to smoke		X				X	
Any smoking in last seven days					X	X	X
Any smoking since Quit date					X	X	X
Lapse and relapse back to smoking					X	X	X
Biochemical verification in quitters^e^							X
**Follow-up details**							
Quit date verified				X			
Contact details^f^			X	X	X	X	X
Treatment allocation			X				
Date products delivered			X				
**Use of other cessation methods**							
Type of cessation method used					X	X	X
**Other outcomes**							
Acceptability						X	
Medication use and compliance				X	X	X	
Crossover				X	X	X	X
Health-related quality of life		X				X	X
Changes to e-cigarette^a^				X	X	X	X
Dual use					X	X	X
Continuation of allocated treatment							X
Adverse events^c^				X	X	X	X
Serious adverse events^c^				X	X	X	X
Cost information							X

*QD* Quit Date

^a^ In those allocated e-cigarettes

^b^ Or pack size, and how long each pack lasts, in people who smoke roll-your-own tobacco

^c^ Separate adverse event form completed

^d^ Separate medication form completed

^e^ A face-to-face meeting to verify quitting status at six months, in those that state they are smokefree

^f^ Separate contact detail form collected

### Primary outcome

The primary outcome measure is six-month CA (Russell Standard) defined as self-report of smoking not more than five cigarettes from the Quit date, supported by biochemical validation [30, 31]. Biochemical validation of self-reported cessation for participants will involve a research assistant or a community smoking cessation provider visiting participants within 72 h if they claim to be abstinent, to obtain an expired air carbon monoxide (CO) reading using a Bedfont Smokerlyser CO Monitor. A reading of ≤ 9 ppm will signify abstinence.

### Secondary outcomes

Secondary outcome measures will be assessed at one, three, six, and (in a subsample) 12 months post-quit date, and are outlined below and in Table 1. Follow-up of the full sample out to 12 months is not possible given the time and budget restrictions associated with the grant funding for the trial.

- CA: The proportion of participants that have stopped smoking, defined as self-report of smoking not more than five cigarettes from the Quit date.
- 7-day point prevalence: The proportion of participants that have stopped smoking, defined as self-report of having smoked no cigarettes (not even a puff) in the past seven days.
- Change in mean number of cigarettes smoked per day: If the participant is still smoking.
- Proportion of participants who have significantly reduced their smoking: Defined as reducing consumption by at least 25% (in terms of number of cigarettes per day).
- Relapse/Lapse: Time to first lapse will be defined as time to the first cigarette smoked from the quit date, even a single puff. Time to first relapse will be defined as time to smoke more than five cigarettes a day for three or more days in a row.
- Use of any other smoking cessation methods: NRT and other nicotine products, and non-NRT methods of cessation such as Zyban (bupropion), clonidine, nortriptyline, varenicline, acupuncture, Quitline etc.
- HRQoL: measured using the NZ EQ-5D Tariff 2 [28].
- Treatment use and compliance: Use of their allocated product including daily use (and reasons for not using daily), and number of pills or pods remaining. For participants allocated to cytisine, compliance will be defined as having taken ≥ 80% of the required number of tablets over the three-month intervention period.
- Signs and symptoms of nicotine withdrawal and urge to smoke: Measured using the MPSS [29].
- Crossover: Participants in the cytisine-only group will be asked whether they accessed and used an e-cigarette (with or without nicotine) during the trial. If they did, they will be asked at what time during the trial, what type of e-cigarette it was, what the nicotine strength was, and what flavour(s) they used. Similarly, participants in the e-cigarette group will be asked whether they accessed and used cytisine during the trial. If they did, they will be asked at what time during the trial, and how much they used.
- Change in e-cigarette use: Participants allocated to the e-cigarette groups will be asked whether they changed the type of e-cigarette device and/or the nicotine strength and/or flavour they used in the e-cigarettes provided. If they did, they will be asked when they did this, and what the device type, nicotine strength and/or flavour was.
- Dual use: Defined as daily use of both their allocated treatment and daily smoking of cigarettes.
- Continuation of product use: Defined as continued use of their allocated treatment after the end of the three-month treatment period.
- Acceptability of the product: Participants will be asked for their views on the use of their allocated product as a cessation aid.
- Recommendations: Participants will be asked whether they would recommend their allocated treatment to others who wanted to quit smoking.
- AEs/Serious AEs: Participants will be asked about any new, unusual, unexpected health events during or since starting treatment, and whether they felt such events were related to treatment (Table 2). Adverse events will be coded using the Medical Dictionary for Regulatory Activities (MedDRA: https://www.meddra.org/).
- Change in weight and body mass index: Self-reported weight to determine change in body mass index from baseline.
- Change in general health: Change from baseline in shortness of breath, cough, asthma, chronic pain, COPD, and mental health.
- Concomitant medication: Other medications being used, including over-the-counter medication.
- Cost outcomes: Cost-per-quitter, cost-per-person reducing their daily cigarette consumption, and the incremental cost-effectiveness ratio.

**Table 2:**
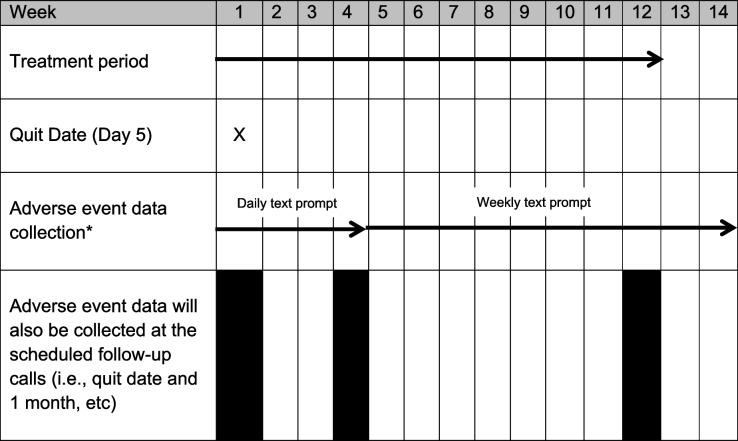
Schedule for adverse event data collection in first 14 weeks

^*^Automated texts, to prompt completion of a web-based adverse event diary, will be sent daily to participants (for the first four weeks), then weekly until week 14 as a prompt to complete the diary. Texts can be received even if the phone has no credit

Should participants require discontinuation of trial treatment, or if they elect to stop taking treatment, follow-up data collection calls will continue as scheduled. Participants may have the trial treatment withdrawn if: they make a voluntary decision to withdraw from follow-up or treatment; the trial medical doctors believe there is significant treatment intolerance and/or it’s in the best interests of the participant; and/or the participant becomes pregnant and/or starts breastfeeding. All women who are pregnant at follow-up will be asked to discuss on-going smoking cessation support with their GP/lead maternity caregiver, and will be withdrawn from the trial. Furthermore, all participants who withdraw or are still smoking at the end of the trial will be referred to smoking cessation support services of their choice.

### Sample size

For 90% power (*p* = 0.05), a sample size of 800 is required (*N* = 300 in the cytisine plus nicotine e-cigarette group, *N* = 300 in the cytisine group, and *N*= 200 in the nicotine e-cigarette group). This sample size is large enough to observe an absolute difference of 13% in smoking abstinence at six months, between the combination and cytisine-only group, and 16% difference between the combination and the nicotine e-cigarette only group (taking account of multiple testing). The predicted difference is based on trial evidence for six month verified CA quit rates of 9% for nicotine e-cigarettes [21, 32], 12% for cytisine [9], and 25% for combination cessation treatment (averaged) [5, 10]. The sample size accounts for a 28% loss-to-follow-up at six months, based on averaged findings from two previous community-based NZ e-cigarette trials [7, 21].

### Data management

Research Electronic Data Capture (REDCap) will be used to collect and manage trial data [33]. A person independent to the trial will be appointed to monitor trial conduct, with monitoring undertaken three times (early in the trial, midway through, and at close-out). Participants’ privacy and confidentiality will be respected through the protection of their data as outlined in the New Zealand Privacy Act (2020, Part 3). An independent DSMC will be established, with clear terms of reference (available upon request). Members of the committee will have no conflicts of interest and will not be directly involved with the trial.

### Statistical analysis

All statistical analyses will be performed using SAS version 9.4 (SAS Institute Inc. Cary NC), and R. No interim analyses are planned. The Statistical Analysis Plan has been pre-registered, and was placed on the trial registry before the first participant was randomised. Demographics, smoking history and related information, cigarette dependence, general health, and alcohol use will be summarised by group and descriptive summary statistics provided. Since any differences between randomised groups at baseline could only have occurred by chance, no formal significance testing will be conducted.

All analyses will be conducted for the following comparisons, 1) cytisine plus nicotine e-cigarettes vs cytisine, and 2) cytisine plus nicotine e-cigarettes vs nicotine e-cigarettes. The main analyses will be carried out on an intention-to-treat basis, with multiple imputation analysis performed to account for missing data using the fully conditional specification logistic regression method (it will be assumed that data are missing at random). Fifty multiple imputed datasets will be created, and the imputation model will include baseline age, sex, and treatment group. The imputed datasets will be analysed using log-binomial regression and combined to output one inference. Incidence rates, risk difference, relative risk and 95% CI will be calculated.

To check robustness of the primary outcome various sensitivity analyses will be undertaken, including analyses addressing the impact of different limits for CO measurements (i.e., at ≤ 3 ppm, ≤ 5 ppm and ≤ 8 ppm), given lack of consensus about the best reading to use [30, 31]. Groups will be compared using chi-squared tests for the primary outcome for the following analyses: assuming participants with missing smoking status data still smoke, complete case analysis, and per protocol analysis (excluding participants with major protocol violations). The consistency of effects for gender, age, ethnicity, education, alcohol consumption, cannabis use, motivation to quit, type of cigarettes smoked, level of behavioural support received, and level of nicotine dependence will be assessed using tests for heterogeneity for the primary outcome. Subgroup analyses will also be conducted by batch number of product received to assess if there were any differences by batch.

Repeated measures mixed models will be used to analyse changes in weight, BMI and mean number of cigarettes over the course of the trial. Several closed- and open-ended questions will be asked about user acceptability, and resulting free text data will be collated, sorted and analysed for key themes using the general inductive method. If combination treatment is shown to be better than monotherapy, a cost-effectiveness analysis will be undertaken to estimate the marginal cost per quitter. The trial will also include a cost utility analysis.

## Discussion

**Fig. 1:**
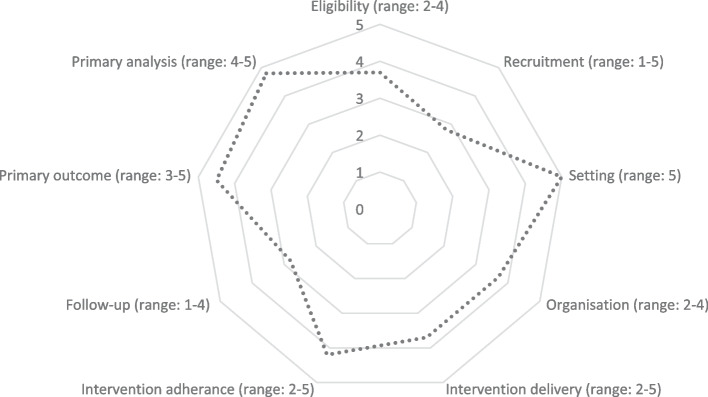
Design of the trial considering the ‘pragmatic to explanatory’ continuum

The paper reports on protocol version 1.0, 18 March 2022. The trial design has been peer reviewed as part of the funding process. The first participant was randomised in May 2022, with recruitment ongoing. The last follow-up visit is estimated to be undertaken in May 2024, with trial findings available by September 2024. The trial has been designed to be as pragmatic as possible, so that the findings are broadly generalisable to NZ. A visual tool (a PRECIS-2 wheel) [34] has been used to show how pragmatic or explanatory the trial design is (Fig. 1). All authors independently assessed nine elements of the trial design, namely participant eligibility, participant recruitment, setting, organisation (the expertise and resources needed to deliver the intervention), intervention delivery (how the intervention should be delivered), adherence to the intervention, follow-up (how closely participants are followed), primary outcome (relevance to participants), and primary analysis (the extent that all data are analysed) [35]. Authors were provided with the PRECIS-2 wheel publication to read, and asked to independently score each element using a Likert scale, which ranged from 1–5, where 1 = ‘very explanatory’ and 5 = ‘very pragmatic’. Authors were then shown deidentified scores and justifications for all investigators, and were offered the chance to rescore. Averaged scores and range (in brackets) are shown in Fig. 1—the more open the radar plot the more pragmatic the trial is.

The two elements of the PRECIS-2 wheel with the greatest score variability between investigators where recruitment and follow-up. For the theme of ‘recruitment’, lower scores related to the fact that advertising is the main method of recruitment, yet if cytisine became available in NZ it would likely be on prescription or sold over the counter in pharmacies (so recruitment processes don’t match likely access in the future). Conversely comments related to higher scores argued that access to cytisine in Canada and Europe is over-the-counter and online (with online advertising). Furthermore, e-cigarettes in NZ are readily available online and in shops (supermarkets, specialist shops) with online advertising – thus recruitment processes match access in NZ and some other countries). For the theme of ‘follow-up’, lower scores related to there being more contact than usual for this trial. People in the real world, who purchase cytisine or e-cigarettes, receive no follow-up, unless these products are accessed via a health professional/pharmacist—which presents an opportunity for follow-up at the next scheduled/unscheduled visit. Conversely comments related to higher scores argued that the behavioural support offered in the trial mimics that offered through NZ’s Quitline text messaging programme.

In considering the design, readers should note that most people trying to quit smoking in NZ receive little personalised support: in-person smoking cessation clinics are uncommon, engagement with the national Quitline is not high, and clinician support is restricted to those who can afford to visit a GP (17% of NZ people ≥ 15 years who smoke daily, live in the poorest neighbourhoods) [1]. Except for biochemical verification of quitting at six months, trial participants are not seen, and receive no reimbursement for their time (although the trial intervention is provided at no cost).

The addition of a text-only group, 12 month follow-up of all participants, equal explanatory power for indigenous Māori, and the use of Ecological Momentary Assessments for data collection would have added value to the trial, but the study design was constrained by the available budget (~ NZ$700,000 for three years: equivalent to ~ US$431,830 or €395,780 as at 26/06/2023). A cluster-randomised trial would have been more appropriate design, given our previous smoking cessation trials found 41–58% of participants live with other people who smoke [7, 8, 21]. However, a cluster trial design would have required a larger sample size, which was not manageable within our available budget.

If the trial hypothesis is proven, the findings will help ensure greater uptake of the tested interventions, through incorporation of the evidence into treatment guidelines and relevant Cochrane Reviews, and the acceptance/ approval of nicotine e-cigarettes and/or cytisine for use in more countries. Greater uptake of these treatments will lead to health gain for people who smoke and quit, their family, and the wider community; a reduction in health inequities; and a reduction in the demand for treatment of tobacco-related diseases by healthcare providers/funders.

## Data Availability

Not applicable.

## Abbreviations

AUDIT-C: Alcohol Use Disorders Identification Test
CA: Continuous abstinence
CI: Confidence Interval
CO: Carbon monoxide
COPD: Chronic Obstructive Pulmonary Disease
EQ-5D: EuroQoL
HRQoL: Health related quality of life
5-HT_3_: 5-Hydroxytryptamine receptors
GP: General Practitioner
IRR: Incidence Rate Ratio
MedDRA: Medical Dictionary for Regulatory Activities
MPSS: Mood and Physical Symptoms Scale
nAChRs: Nicotinic acetylcholine receptors
NRT: Nicotine Replacement Therapy
NZ: New Zealand
PRECIS-2: PRagmatic-Explanatory Continuum Indicator Summary-2
QD: Quit date
RR: Relative Risk
TBS: Text-based behavioural support
