# Correction: Effectiveness of nicotine salt vapes, cytisine, and a combination of these products, for smoking cessation in New Zealand: protocol for a three-arm, pragmatic, community-based randomised controlled trial

**DOI:** 10.1186/s12889-025-21438-8

**Published:** 2025-01-22

**Authors:** Natalie Walker, Amanda Calder, Joanne Barnes, George Laking, Varsha Parag, Chris Bullen

**Affiliations:** 1 School of Population Health, National Institute for Health Innovation, The University of Auckland, Private Bag 92019, Auckland, 1142 New Zealand; 2 School of Pharmacy, The University of Auckland, Private Bag 92019, Auckland, 1142 New Zealand; 3 Department of Molecular Medicine and Pathology, The University of Auckland, Private Bag 92019, Auckland, 1142 New Zealand

**Correction: BMC Public Health 23, 1760 (2023)**

**https://doi.org/10.1186/s12889-023-16665-w**

The original publication of this article was missing 1 exclusion criteria in the eligibility section:

- History of severe allergy and/or poorly controlled asthma

The original publication has been updated to list this exclusion criteria. This does not change the results nor conclusions of the article. The original publication has been updated.
